# The role of gut leakage and immune cell miss-homing on gut dysbiosis-induced lung inflammation in a DSS mice model

**DOI:** 10.1371/journal.pone.0324230

**Published:** 2025-05-28

**Authors:** Mariam Wed Eladham, Narjes Saheb Sharif-Askari, Priyadharshini Sekar, Bushra Mdkhana, Balachandar Selvakumar, Baraa Khalid Salah Al-Sheakly, Fatemeh Saheb Sharif-Askari, Ibrahim Hachim, Rabih Halwani

**Affiliations:** 1 Research Institute for Medical and Health Sciences, University of Sharjah, Sharjah, United Arab Emirates; 2 Department of Clinical Sciences, College of Medicine, University of Sharjah, Sharjah, United Arab Emirates; 3 Department of Pharmacy Practice and Pharmacotherapeutics, College of Pharmacy, University of Sharjah, Sharjah, United Arab Emirates; 4 Prince Abdullah Ben Khaled Celiac Disease Chair, Department of Pediatrics, Faculty of Medicine, King Saud University, Riyadh, Saudi Arabia; Cincinnati Children's Hospital Medical Center, UNITED STATES OF AMERICA

## Abstract

**Background:**

Inflammatory Bowel Disease (IBD), encompassing Crohn’s disease and ulcerative colitis, affects millions globally, with extraintestinal manifestations (EIMs) occurring in 25–40% of patients. Among these, respiratory complications are of particular concern, yet the immunologic and physiologic mechanisms underlying gut-lung interactions remain poorly understood. The gut-lung axis (GLA) describes bi-directional communication between the gut and lungs, where microbial dysbiosis in the gut can drive lung inflammation and immune dysregulation.

**Methods:**

Mice were treated with 4% DSS for 7 days to induce colitis. Gut permeability, tight junction protein expression, lung inflammation, immune cell trafficking, and microbial translocation were assessed through histology, qPCR, flow cytometry, and GFP-tagged fecal microbiome experiments.

**Results:**

DSS treatment led to significant disruption of the gut barrier, with upregulation of gut leakage markers and downregulation of tight junction proteins. Lung inflammation was characterized by elevated IL-17, neutrophil infiltration, and airway hyperresponsiveness. Flow cytometry revealed mis-homing of gut-primed immune cells (α4β7+ and CCR9 + CD4+) to the lungs and tracking bacteria via GFP- tagged fecal microbiome confirmed microbial translocation from the gut to the lungs which may contribute to lung inflammation.

**Conclusion:**

Disrupted gut integrity facilitates microbial translocation and immune cell mis-homing, contributing to lung inflammation. These results provide new insights into how gut dysbiosis influences respiratory inflammation.

## 1. Introduction

Inflammatory Bowel Disease (IBD), encompassing Crohn’s disease and ulcerative colitis, affect millions of people in Europe, North America, and Australasia, leading to a substantial disease burden [1]. However, it is increasingly recognized that IBD has systemic implications, with extraintestinal manifestations (EIMs) occurring in nearly 25–40% of patients. These manifestations often involve the skin, joints, eyes, and lungs, among other organs [2]. Evidence for EIMs of IBD is known and emerging, however, little is known about immunologic and physiologic mechanism. Understanding the intricate interplay between the gut and lung, known as the gut-lung axis (GLA) is crucial for elucidating the mechanisms underlying these pathologies. The GLA indicates the presence of a mechanism where bi-directional interplay occurs between the gut and its associated immune system and the lungs and its immune system [3]. The GLA is regulated by several potential mechanisms proposed recently in current literature [4].

Gut leakage facilitates the translocation of microbial products, such as lipopolysaccharides (LPS) and peptidoglycans, into systemic circulation. Microbial leakage activates systemic immune responses, inducing inflammation in distal organs, including the lungs [5]. Recent studies are increasingly linking gut microbiota dysbiosis to diseases such as asthma and chronic obstructive pulmonary disease (COPD) [6,7].

The gut microbiota plays a crucial role in maintaining immune balance by regulating the equilibrium between pro-inflammatory Th17 cells and anti-inflammatory Treg cells within the gastrointestinal tract [8]. Disruption of this microbiome balance has been associated with an increased presence of CD4 + IL-17-producing cells in the gut [9]. Notably, the immune-regulatory effects of the microbiota extend beyond the local intestinal environment to influence peripheral organs such as the brain [10,11], liver [12], and lungs [13,14]. In ischemic conditions like mesenteric ischemia, hypoxia damages the gut barrier, increasing permeability and enabling gut bacteria and endotoxins (e.g., LPS) to enter the bloodstream. This triggers systemic inflammation as these microbes reach the lungs, activating immune cells and elevating cytokines such as TNF-α and IL-6. The resulting inflammatory cascade contributes to acute lung injury (ALI) or acute respiratory distress syndrome (ARDS). Ischemia-induced gut dysbiosis exacerbates bacterial overgrowth and translocation, creating a feedback loop that links gut and lung dysfunction [15]. Similarly, severe traumatic brain injury (TBI) disrupts the gut-brain axis, weakening the gut barrier through systemic inflammation, stress hormones, and oxidative stress. This allows gut bacteria and endotoxins like LPS to enter the bloodstream and disseminate to organs like the lungs, where they trigger inflammatory responses, contributing to ALI or ARDS. TBI-induced gut motility and immune dysregulation further heighten bacterial translocation, creating a feedback loop that worsens brain injury and hinders recovery [16]. This systemic impact is mediated through the migration of T cells [17,18], highlighting a critical connection within the gut and extraintestinal organs.

Gut-homing receptors (such as CCR9 and α4β7) are surface molecules expressed on immune cells, such as T cells, that facilitate their migration to and retention within the gastrointestinal tract upon binding to their CCL25 and MadCAM-1 (Mucosal Addressin Cell Adhesion Molecule-1) ligands respectively [19,20]. In normal conditions, α4β7 expression is primarily associated with immune cells that traffic to the gut mucosa. These immune cells, particularly T cells, express α4β7 integrin along with the gut-specific addressin MAdCAM-1 facilitating their homing to the gut-associated lymphoid tissue (GALT) and intestinal mucosa [21]. Chronic inflammation alters immune cell homing patterns, leading to the migration of gut-primed lymphocytes to extraintestinal sites [18]. This mis-homing mechanism underscores how intestinal inflammation in IBD could instigate or worsen pulmonary conditions. Therefore, highlighting the significance of gut barrier integrity and immune cell trafficking in modulating lung immune responses is needed.

The gut-lung axis represents a critical but understudied pathway in understanding EIMs of IBD. This study aims to elucidate the mechanisms by which intestinal barrier dysfunction, immune cell trafficking and bacterial translocation contribute to lung inflammation in a dextran sulfate sodium (DSS)-induced colitis mouse model. In addition, we also investigated on the mis-homing of immune cells into lungs during the IBD. Our current study shed light on the mechanisms observed in pulmonary inflammation contributing to a deeper understanding of the gut-lung axis and its role in respiratory complications.

## 2. Methods

### 2.1. DSS-induced colitis mouse model

Six- to eight-week-old female C57BL/6 mice (18-20g) were obtained from the animal facility at the University of Sharjah. All mice were housed in pathogen-free conditions within sterile, individually ventilated cages (TECNIPLAST). They were kept on a 12-hour light-dark cycle and provided with a sterile maintenance diet (Altromin 1324 TPF) and sterile distilled water. Autoclaved, dust-free aspen bedding (ABEDD) was used for environmental enrichment.

**Fig 1:**
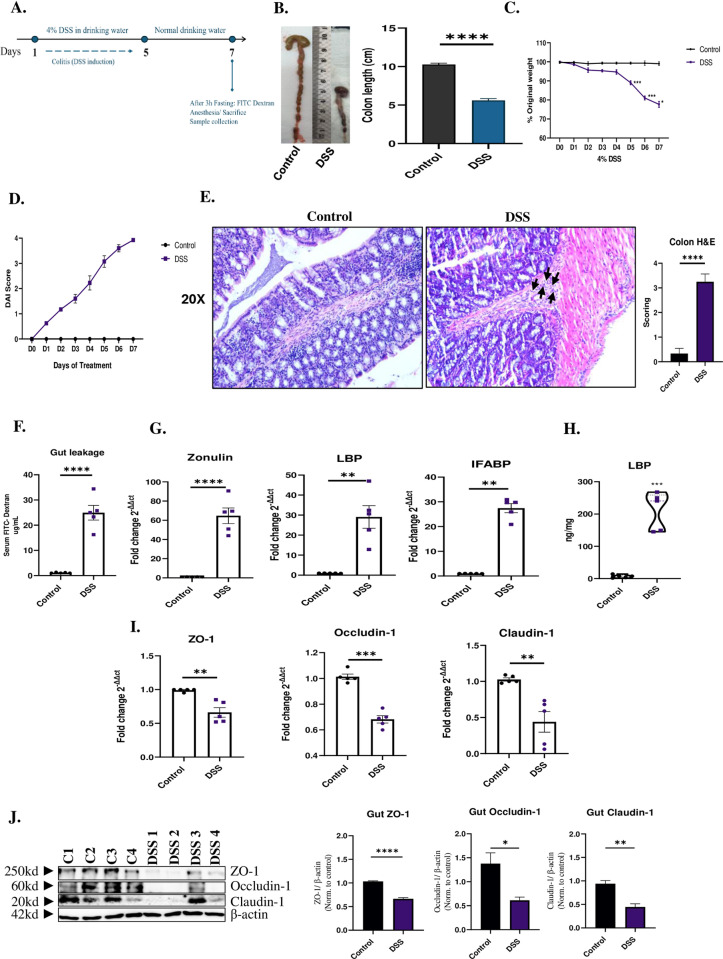
Decreased expression of tight junction proteins and disruption of intestinal barrier integrity in DSS-induced colitis mouse model. Mice were treated with 4% DSS in drinking water for 5 days, followed by 2 days of regular water, to induce acute colitis. **(A)** Schematic timeline of DSS administration and assessments. **(B)** Colon length was significantly shorter in DSS-treated mice compared to controls. **(C)** Body weight loss was observed in DSS-treated mice starting from day 4 and progressively worsened by day 7. **(D)** Disease Activity Index (DAI) scores indicated an increase in colitis severity in DSS-treated mice. **(E)** H&E-stained histology slides at 20X and inflammation score colon tissues. **(F)** Gut permeability assay measuring serum FITC-dextran levels in DSS-treated and control mice. **(G)** qPCR analysis of mRNA expression of gut leakage markers; Zonulin, LBP, and IFABP in DSS-treated and control mice. **(H)** ELISA analysis of serum LBP levels in DSS-treated and control mice. **(I)** qPCR analysis of tight junction-related genes ZO-1, Occludin-1, and Claudin-1 in DSS-treated and control mice. **(J)** Protein levels of tight junction markers assessed in DSS-treated and control mice groups. Data representative from n = 5 mice in each group. Full blots are supplemented in **Fig A** in S1 Raw images. The values were compared across the different groups using an independent student’s t-test between the groups. Results are presented as mean (± SEM) and relative to control mice. *p < 0.05, **p < 0.01, ***p < 0.001, ****p < 0.0001 compared to control mice.

Briefly, colitis model was induced via oral administration of dextran sulfate sodium (DSS; MW 36–50 kDa, TCI, D5144) as previously described [22]. After acclimation, mice were randomly divided into two groups (n = 5–6 per group): a negative control group and a DSS-treated group. Gut inflammation was induced by treating with 4% (w/v) DSS in drinking water ad libitum for 5 days, followed by a DSS-free period of 2 days, concluding on day 7 as shown in (Fig 1A). Control group mice received normal drinking water throughout the experiment. Body weight, stool consistency, and the presence of blood in the rectum or stool were monitored and recorded daily. The Disease Activity Index (DAI) was calculated following a previously established method [23], using the formula: Total Score = (Body Weight Loss + Stool Consistency + Rectal Bleeding)/ 3. This composite score was used to assess the severity of the disease in each mouse.

### 2.2. Ethics statement

All procedures were performed in compliance with protocols approved by the Animal Care and Use Committee of the University of Sharjah (Approval No. ACUC-06-02-2024). At the end of the experiments, mice were anesthetized with isoflurane inhalation before being euthanized by cervical dislocation, with all efforts made to minimize suffering.

### 2.3. Airway hyperresponsiveness (AHR)

Airway hyperresponsiveness (AHR) was assessed by measuring total lung resistance (Rrs, cm H₂O·s/mL) using the FlexiVent system (SCIREQ, Montreal, Canada). Mice were anesthetized via intraperitoneal injection of ketamine (114.5 mg/kg) and xylazine (6.9 mg/kg) [24], followed by a tracheostomy using a stainless-steel cannula. Nebulization with escalating doses of methylcholine (0–50 mg/mL, MCh, Sigma) was performed. Airway resistance was calculated and recorded in cm H₂O·s/mL using FlexiVent software (version 8.0.4).

### 2.4. Dextran- FITC assay

Gut permeability was determined by fluorescein isothiocyanate-dextran dextran (FITC-dextran) assay. FITC-dextran (Sigma-Aldrich, St. Louis, MO, USA) (80 mg/ml) was orally administered to mice 3h before sacrifice. Blood samples were collected via the inferior vena cava and the concentration of FITC-dextran was measured to assess intestinal permeability. FITC-dextran was measured in serum with fluorospectrometric at 485nm and 528nm excitation and emission wavelength [25].

### 2.5. Western blot

Proteins from gut homogenate were extracted using RIPA lysis buffer, supplemented with 1 mM phenylmethylsulfonyl fluoride (PMSF) and a 1x Protease Inhibitor Cocktail (both from Sigma-Aldrich, Germany). The resulting protein lysates were then collected and quantified utilizing the ThermoScientific Pierce BCA Protein Assay Kit (ThermoFisher Scientific, US). Subsequently, the proteins were separated on a 10% SDS-polyacrylamide gel and transferred onto nitrocellulose membranes using a semi-dry transfer system. The blocking of membranes was made with 5% BSA prepared in Tris-Buffered Tween saline for 1hr and incubated with primary anti- ZO-1 (cat. #**8193**, 1:1000), anti- Claudin1 (cat. #**13255,** 1:1000), anti- Occludin1 (cat. #**91131**, 1:1000) or anti-βactin (1:1000) antibodies (Cell Signaling Technologies, Danvers, MA) at 4^º^C overnight. The following day, secondary anti-rabbit IgG (1:1000) antibodies conjugated to horseradish peroxidase were incubated for 1 h at room temperature. Sapphire™ NIR-Q Biomolecular Imager (Azure Biosystems, Dublin, US) was used to detect the protein bands, which were subsequently quantified using ImageJ software.

### 2.6. Histopathological analyses

Colon and lung tissues were fixed in 10% formalin, embedded in paraffin blocks, and stained with hematoxylin and eosin (H&E) or periodic acid-Schiff (PAS) using a PAS kit (Sigma Aldrich) according to the manufacturer’s instructions. H&E and PAS staining were evaluated and scored based on a modified scoring system described in previous studies [26,27].

To assess the degree of congestion, intra-alveolar haemorrhage, inflammatory cell infiltration was quantified across various **lung** sections. The scoring criteria were as follows:

- **Score 0:** no evidence of inflammatory infiltrate, congestion, intra-alveolar haemorrhage.
- **Score 1:** mild congestion and intra-alveolar haemorrhage.
- **Score 2:** mild-moderate congestion and intra-alveolar haemorrhage.
- **Score 3:** moderate congestion and moderate intra-alveolar haemorrhage, evidence of inflammatory infiltrate.
- **Score 4:** severe congestion and severe intra-alveolar haemorrhage with severe inflammation.

PAS staining was used to assess mucus production, with the percentage of PAS-positive mucus-containing cells quantified relative to the total number of epithelial cells to evaluate goblet cell hyperplasia [26–29].

To assess morphology, leukocytes infiltrate, goblet cells were quantified across various **colon** sections. The scoring criteria were based on a modified scoring system described in previous studies [30]:

- **Score 0:** normal morphology, no evidence of leukocytes infiltrate, normal goblet cells, normal crypts.
- **Score 1:** mild infiltration of leukocytes, mild reduction in goblet cells, and mild tissue injury.
- **Score 2:** mild- moderate infiltration of leukocytes, mild-moderate reduction in goblet cells and mild- moderate tissue injury.
- **Score 3:** moderate infiltration of leukocytes, moderate reduction in goblet cells and moderate tissue injury.
**• Score 4:** extensive infiltration reaching to the submucosa in some areas, severe reduction in goblet cells in some areas and severe tissue injury.

### 2.7. Quantitative real-time polymerase chain reaction (qRT-PCR)

Total RNA/ plasmid DNA was extracted from lung tissues using the Tiazol/chloroform extraction method. RNA concentrations were quantified using a NanoDrop 2000 spectrophotometer (Thermo Scientific, USA). cDNA synthesis was performed using the High-Capacity cDNA Reverse Transcription Kit (Thermo Scientific, USA).

Real-time PCR was conducted to quantify the expression levels of tight junction markers (ZO-1, Claudin-1, Occludin-1), gut leakage markers (IFABP, LBP, Zonulin), inflammatory markers, gut homing receptors and ligands (CCL25, MadCam-1) and GFP with β-actin serving as the housekeeping gene. The primers listed in **Table 1** were designed specifically for this study using NCBI- Primer Blast and validated through in silico PCR to confirm specificity and efficiency. The reactions were performed on a Quant Studio 5 system (Thermo Fisher) using 5x HOT FIREPol® EvaGreen® qPCR Supermix (Solis BioDyne). All samples were analysed in triplicates. Average threshold cycle (Ct) values were calculated for each gene, and relative expression levels were determined using the 2^(-ΔΔCt) method.

**Table 1:** List of mice primer sequences used in qRT-PCR.

Target genes	Forward 5’-3’	Reverse 5’- 3’
*Zonulin*	CGAATGTGAGGCAGTGTGTG	ATAGAGCCACCGATGATGCG
*LBP*	TGACTACAGTTTGGTGGCGG	GGAGAGCGGTGATTCCGATT
*IFABP*	TGCTGTCCGAGAGGTTTCTG	AGAATCGCTTGGCCTCAACT
*ZO-1*	GCTTCTCTTGCTGGCCCTAA	GGGAGCCTGTAGAGCGTTTT
*Claudin-1*	CAAGGGCCCGCATACTTTCT	TGCTCCGAGACTACCCAAAG
*Ocludin-1*	TCTTTCCTTAGGCGACAGCG	AGATAAGCGAACCTGCCGAG
*IL-4*	GCATTTTGAACGAGGTCACAGG	CTCTCTGTGGTGTTCTTCGTTG
*IL-13*	ACACAAGACCAGACTCCCCT	GGGAATCCAGGGCTACACAG
*Il-17a*	ACCGCAATGAAGACCCTGAT	TCCCTCCGCATTGACACA
*CCL25*	GAATGTGAAGAGGGCGATGA	CTCACGCTTGTACTGTTGGG
*Madcam-1* *GFP*	GAGCCAGACCTCACCTAAGC GGTCCTTCTTGAGTTTGTAAC	GCTGCCAATCCATAGGACGA CCATCTAATTCAACAAGAATTGGGACAAC
*β-actin*	ACCCTAAGGCCAACCGTGA	CAGAGGCATACAGGGACAGCA

### 2.8. ELISA

Inflammatory cytokines (IL-4, IL-13, IL-17) and gut leakage marker (LBP) in serum or BALF was estimated according to manufacturer instructions.

### 2.9. Flow cytometry

To analyze the percentages of eosinophils and neutrophils in the BALF. BALF were collected by washing mouse airways twice with 500 μl PBS-EDTA (0.5nM). Total cells were counted after centrifugation (1500 RPM, 4C, 10 min). The cells were incubated with CD45 APC-Cy7, CD11b PE, Siglec-F APC, CD11c FITC and Ly6G AF 488 (BD Biosciences) anti-mouse antibodies at 4C for 20 min. To evaluate the percentage CD4 + gut homing receptors in gut and lung homogenates, the tissues were enzymatically homogenized with Liberase and DNase I (Roche, Germany) to prepare single-cell suspensions. The single cell suspensions from the two groups were stained with CD3 AF700, CD4 erCp-Cy5.5, CCR9 FITC and LAMP (α4β7) APC (S1A and S1B Fig).

### 2.10. Tracking of bacterial translocation from the gut to lung of mice by GFP- tagged fecal microbiota transplant

#### 2.10.1. Isolation of fecal microbiota.

Stool samples were freshly collected on day 5 from healthy control mice and DSS model mice. Fecal samples were pooled separately for each group and resuspended in sterile 0.9% saline at a ratio of 1:5 (w/v). Fecal microbiota was extracted using a density gradient centrifugation method with Nycodenz reagent (Iohexol; Progen, Cat. No. 18003), following a previously described protocol [31]. The isolated fecal microbiota layer was resuspended in sterile PBS, maintained at 4°C, and immediately used for transformation with GFP-tagged plasmids.

#### 2.10.2. Preparation of GFP-tagged plasmid.

The plasmid pBTK509, a gift from Jeffrey Barrick (Addgene plasmid #110616; RRID:Addgene_110616) [32], and pBbB5a-GFP, a gift from Jay Keasling (Addgene plasmid #35352; RRID:Addgene_35352) [33], were used in this study. Both plasmids were propagated separately in *E. coli* DH5α, cultured at 37°C for 16–18 hours in BHIB supplemented with 100 µg/mL ampicillin. Plasmid DNA was extracted using the Monarch Plasmid Miniprep Kit (Cat. No. T101L), and the concentration of each plasmid was determined using a NanoDrop 2000 spectrophotometer (Thermo Scientific, USA). The extracted plasmids were stored at −20°C until further use.

#### 2.10.3. GFP- tagged plasmid transformation into isolated fecal microbiota.

To 300 µL of isolated fecal microbiota, 100 µL of 0.1 M calcium chloride was added, followed by 30 µL each of pBbB5a-GFP and pBTK509-GFP plasmids. The mixture was placed on ice for 30 minutes, subjected to heat shock at 42°C for 2 minutes, and subsequently returned to ice for 5 minutes. Next, 2 mL of BHIB supplemented with 100 µg/mL ampicillin and 0.5 mM IPTG was added to the suspension, which was incubated at 37°C for 1 hour. After incubation, the GFP-tagged fecal microbiota was prepared and ready for oral administration to mice to facilitate tracking of bacterial translocation from the gut to the lung.

#### 2.10.4. Tracking of bacterial translocation from the gut to lung of mice.

Approximately 100 µL of GFP-tagged fecal microbiota was administered via oral gavage to both control and DSS-treated mice on Day 6. After this period, the mice were euthanized, and their gut and lung tissues were harvested and stored at 4°C until further processing. The harvested tissues were enzymatically homogenized with Liberase and DNase I, and the homogenates were used for detection of GFP-tagged fecal microbiota through CFU counting, qPCR, spectrophotometry, and flow cytometry.

For CFU analysis, the gut and lung homogenates from both control and DSS-treated mice were incubated in BHIB supplemented with 100 µg/mL ampicillin for 3 hours. Following incubation, the mixtures were plated onto BHIA plates containing 100 µg/mL ampicillin and incubated at 37°C for 16–18 hours. Colony counts were recorded.

Plasmid DNA was extracted from the gut and lung homogenates using the Monarch Plasmid Miniprep Kit (Cat. No. T101L). Quantitative PCR (qPCR) was performed on a Quant Studio 5 system (Thermo Fisher Scientific) using 5x HOT FIREPol® EvaGreen® qPCR Supermix (Solis BioDyne) with primers listed in Table 1. GFP fluorescence was quantified at 485nm and 528nm excitation and emission wavelength 475nm. To evaluate the percentage of GFP- tagged fecal microbiome in gut and lung homogenates by flow cytometry, the single-cell suspension was prepared and measured by gating small-sized cells on a logarithmic scale (S1C Fig) using the BD AriaIII flow cytometer (BD-Biosciences).

### 2.11. Statistical analysis

All data are expressed as mean ± standard error of the mean (SEM). An unpaired independent Student’s t-test was used for comparisons between two groups. A p-value less than 0.05 was considered statistically significant. Data analysis was performed using GraphPad Prism software, version 8.4.2 (GraphPad Software, Inc., La Jolla, CA, USA). Statistical significance is represented as follows: *p < 0.05, **p < 0.01, ***p < 0.001, and ****p < 0.0001.

## 3. Results

### 3.1. Decreased expression of tight junction proteins and disruption of intestinal barrier integrity in DSS-Induced colitis mouse model

To establish the DSS-induced colitis model, mice were treated with 4% DSS for 7 days (Fig 1A). DSS-treated mice exhibited significant physiological changes, including a marked reduction in colon length (P < 0.0001) (Fig 1B) and progressive weight loss during treatment (Fig 1C). Furthermore, the Disease Activity Index (DAI) score was significantly elevated in DSS-treated mice (P < 0.0001), indicating severe colonic inflammation (Fig 1D). Histological analysis of colonic tissues using H&E staining revealed substantial mucosal damage in DSS-treated mice (3.3 score vs 0.3; P < 0.0001). Control samples displayed intact crypt architecture and epithelial integrity, whereas DSS-treated colons exhibited crypt distortion, severe inflammatory cell infiltration, and mucosal erosion, indicative of chronic colonic inflammation 7 days post-DSS treatment (Fig 1E).

To assess intestinal barrier integrity, a gut permeability assay was performed by measuring FITC-dextran levels in the serum. DSS-treated mice showed a significant increase in serum FITC-dextran compared to controls, confirming compromised gut barrier function (25 vs 1.1; P** **< 0.0001) (**Fig 1F**). The mRNA expression of gut leakage markers, including Zonulin (64.7 vs 1.1 folds; P** **< 0.0001), LBP (29.1 vs 1 folds; P** **< 0.01), and IFABP (27.46 vs 1.014 folds; P** **< 0.01) was significantly upregulated in DSS-treated mice, as shown by qPCR analysis (**Fig 1G**). This was corroborated by ELISA results, which demonstrated markedly elevated serum LBP levels in the DSS group compared to controls (P** **< 0.001) (**Fig 1H**). Conversely, qPCR analysis of tight junction-related genes revealed a significant downregulation in ZO-1 (0.7 vs 1 folds; P** **< 0.01), Occludin-1 (0.7 vs 1 folds; P** **< 0.001), and Claudin-1 (0.4 vs 1.0 folds; P** **< 0.01) in DSS-treated mice compared to control mice (**Fig 1I**). In alignment with mRNA findings, a notable decrease in the protein levels of these tight junction markers (**Fig 1J**). These findings further confirmed the disruption of intestinal tight junctions and impaired barrier integrity in DSS-induced colitis.

### 3.2. DSS-Induced Colitis is associated with lung inflammation and IL-17 upregulation in mice

**Fig 2:**
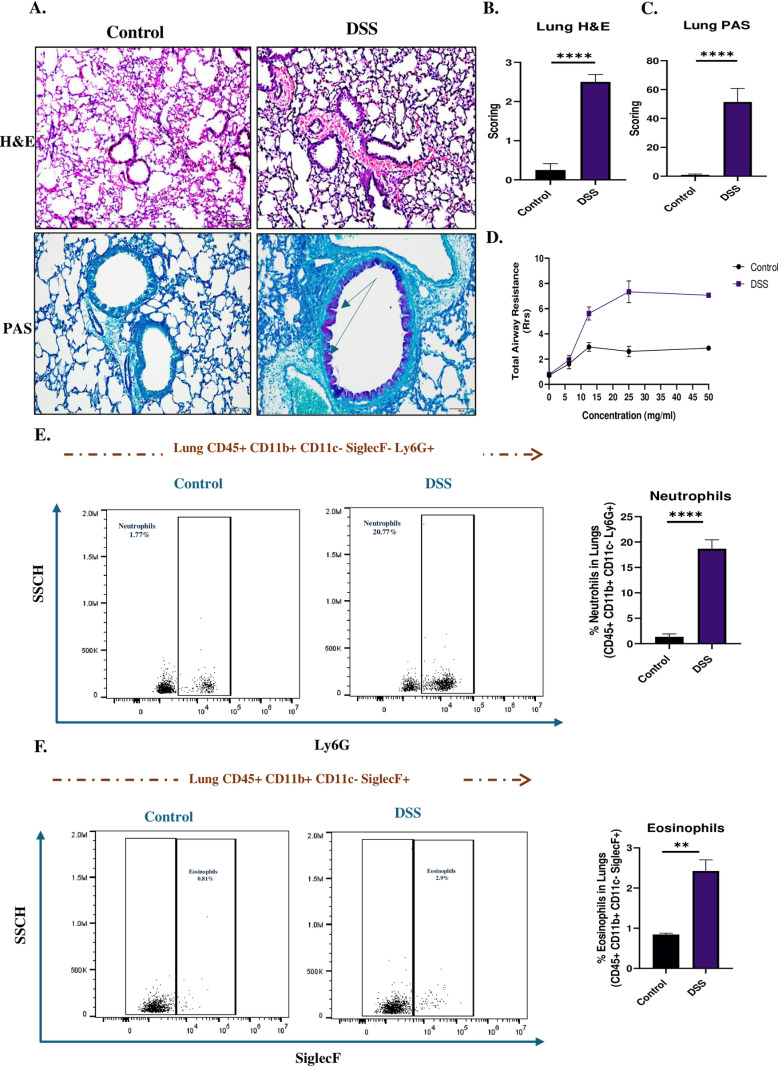
DSS-Induced Colitis is associated with lung inflammation and IL-17 upregulation in mice. **(A)** Histological analysis of H&E and PAS-stained lung sections from DSS-treated and control mice, showing lung inflammation and mucus hypersecretion. **(B)** Inflammation scoring of H&E-stained lung sections. **(C)** Mucus production scoring of PAS-stained lung sections. **(D)** Airway hyperresponsiveness assessed by total airway resistance. **(E)** Flow cytometry analysis of neutrophil infiltration in lung tissues. **(F)** Flow cytometry analysis of eosinophil infiltration in lung tissues. Data representative from n = 5-6 mice in each group. Results are presented as mean (± SEM) and were compared using an independent student’s t-test. *P < 0.05, **P < 0.01, ***P < 0.001, ****P < 0.0001 compared to control mice.

Lung inflammation was induced in DSS-treated mice and confirmed by histopathological and immunohistochemical analyses. H&E staining of lung tissues revealed inflammatory cell infiltration and tissue remodelling, which were further corroborated by PAS staining, indicating mucus hypersecretion in the airways (Fig 2A). Scoring of H&E and PAS-stained sections demonstrated a significant increase in inflammation and mucus production in the DSS group compared to control (2.5 vs 0.3 scores; P < 0.0001) and (51.4 vs 0.9 scores; P < 0.0001) (Fig 2B and 2C). Additionally, airway hyperresponsiveness was evaluated, and DSS-treated mice displayed a significant increase in total airway resistance (P < 0.0001), indicating impaired lung function (Fig 2D). Flow cytometry analysis further identified a significant increase of neutrophils (P < 0.0001) (Fig 2E and S2A Fig) with mild increase in eosinophils (P < 0.01) (Fig 2F and S2B Fig) into lung tissues of DSS-treated mice, suggesting a skewed Th17 and mild Th2 response.

**Fig 3:**
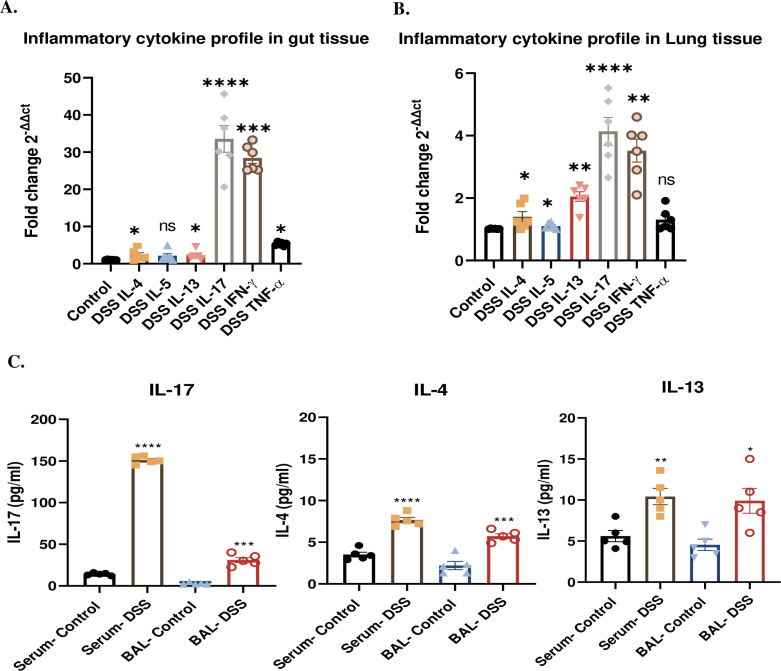
Inflammatory cytokine profile in gut and lungs of DSS-induced colitis mice. **(A)** qPCR analysis of IL-4, IL-5, IL-13 and IL-17 mRNA expression in lung tissues. **(B)** qPCR analysis of IL-4, IL-5, IL-13 and IL-17 mRNA expression in lung tissues. **(C)** ELISA analysis of IL-17, IL-4, and IL-13 protein levels in BAL fluid and serum. Data representative from n = 5-6 mice in each group. Results are presented as mean (± SEM) and were compared using an independent student’s t-test. *P < 0.05, **P < 0.01, ***P < 0.001, ****P < 0.0001 compared to control mice.

One of the hallmarks of colitis-induced mouse model is the increased levels of IL-17 [34]. Therefore, the expression of these mediators along with the other inflammatory cytokines was subsequently examined in gut and lung tissues. The model was presented with a significant increase by 33.6-fold in IL-17 (P < 0.0001), 2.4-fold in IL4 (P** **< 0.05), and 2.3-fold in IL-13 (P** **< 0.05) mRNA expression levels in gut tissues of DSS-treated mice compared to controls (Fig 3A). On the other hand, qPCR analysis of lung tissues revealed a significant upregulation of IL-17, IL4, IL5 and IL13 mRNA by 4.2-fold (P < 0.0001), 1.4-fold (P < 0.05), 1.1-fold (P < 0.05) and 2-fold (P < 0.01) respectively in DSS-treated mice compared to controls, confirming flow cytometry results (Fig 3B). These results confirm that lung inflammation was influenced by gut-derived factors. In addition, protein levels of these inflammatory markers were confirmed by ELISA, to measure IL-17, IL-13 and IL-4 levels in both BAL fluid and serum. A significant upregulation of IL-17 was observed in the DSS-treated group, with a 150-fold increase in serum (P < 0.0001) and 31-fold increase in BAL (P < 0.001), suggesting that systemic circulation may serve as a conduit for gut-derived cytokines. ELISA analysis also revealed upregulation of IL-13 and IL-4, with serum levels increasing by 4.1-fold and 3-fold, and BAL fluid levels rising by 3-fold each in the DSS-treated group compared to controls. (Fig 3C). This mild elevation aligns with the qPCR findings, indicating a subtle Th2 contribution to the lung inflammatory response.

These findings underscore the crucial role of gut-lung crosstalk in inflammation. DSS-induced colitis leads to increased gut permeability, facilitating the translocation of inflammatory mediators like IL-17 into systemic circulation. The elevated IL-17 in serum and BAL fluid strongly suggests that gut inflammation contributes to the amplification of lung inflammation. This systemic cytokine spillover likely triggers neutrophil recruitment, airway remodelling, and hyperresponsiveness in the lungs, emphasizing the importance of addressing gut inflammation as a therapeutic target for managing lung inflammatory conditions.

### 3.3. Miss-homing of gut-primed CD4 T cells into lungs of DSS mice

**Fig 4:**
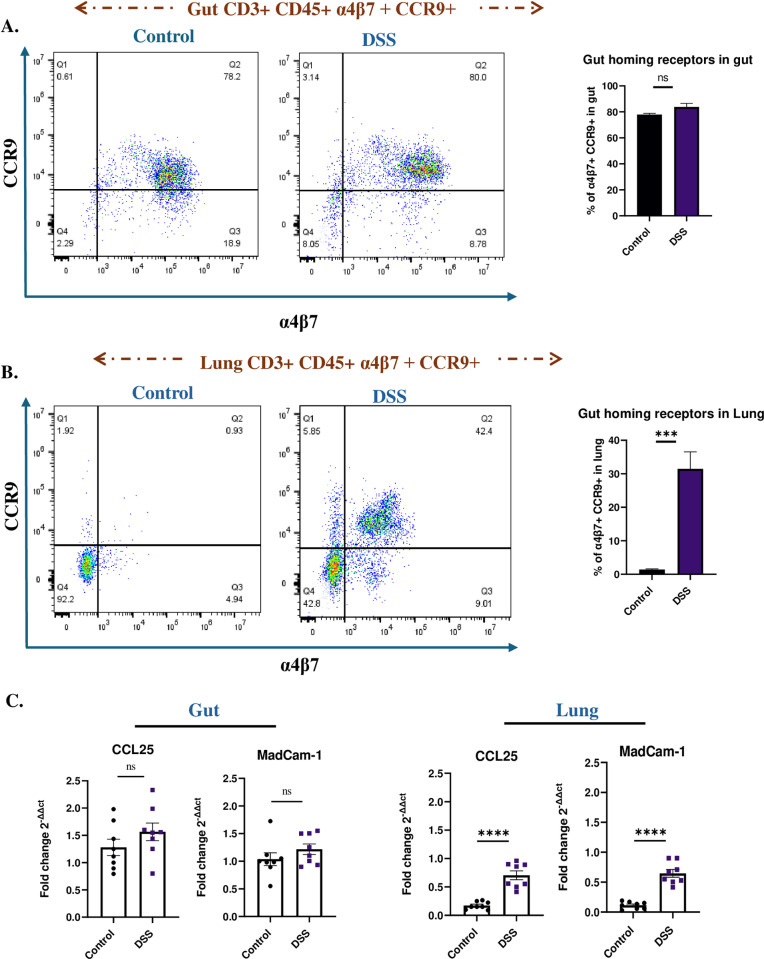
Miss-homing of gut-primed CD4 T cells into lungs of DSS mice. **(A)** Flow cytometry analysis of α4β7+ and CCR9 + CD4 + cells from gut tissues in control and DSS-treated mice. **(B)** Flow cytometry analysis of α4β7+ and CCR9 + CD4 + cells from lung tissues in control and DSS-treated mice. **(C)** qPCR analysis of gut homing ligands CCL25 and MadCam-1 in gur and lungs of control and DSS-treated mice. Data representative from n = 5 mice in each group. Results are presented as mean (± SEM) and were compared using an independent student’s t-test. *P < 0.05, **P < 0.01, ***P < 0.001, ****P < 0.0001 compared to control mice.

To further investigate the mechanism of gut leakage associated lung inflammation, we evaluated the expression on CD4 T cells of gut-homing receptors α4β7 and CCR9 in both the gut and lung tissues of DSS-treated and control mice. In the gut, both α4β7 and CCR9 are essential for the homing of T lymphocytes to mucosal sites, for immune surveillance and gut homeostasis [35]. However, in lung lymphocytes, these receptors are not typically expressed under normal conditions [4]. Flow cytometry analysis on CD4 + cells from gut tissues showed no significant difference in the expressions of α4β7 and CCR9 in control and DSS-treated mice (Fig 4A and S3A Fig). However, in the lungs of DSS-induced colitis mice both α4β7+ and CCR9 + CD4 + cells were significantly upregulated in the lung tissues (31.5% vs 1.4%; P < 0.001) (Fig 4B and S3B Fig). Furthermore, the mRNA levels of gut homing receptor ligands showed a significant upregulation of CCL25 (P < 0.0001) and MadCam-1 (P < 0.0001) in the lungs of DSS-treated mice relative to the gut of control mice group (Fig 4C). This data suggests a mis-homing of gut homing CD4 + cells and their ligands, into the lungs under conditions of increased intestinal permeability, reinforcing the crosstalk between the gut and lung in inflammatory responses.

### 3.4. Intestinal permeability drives microbial translocation to the lungs

**Fig 5:**
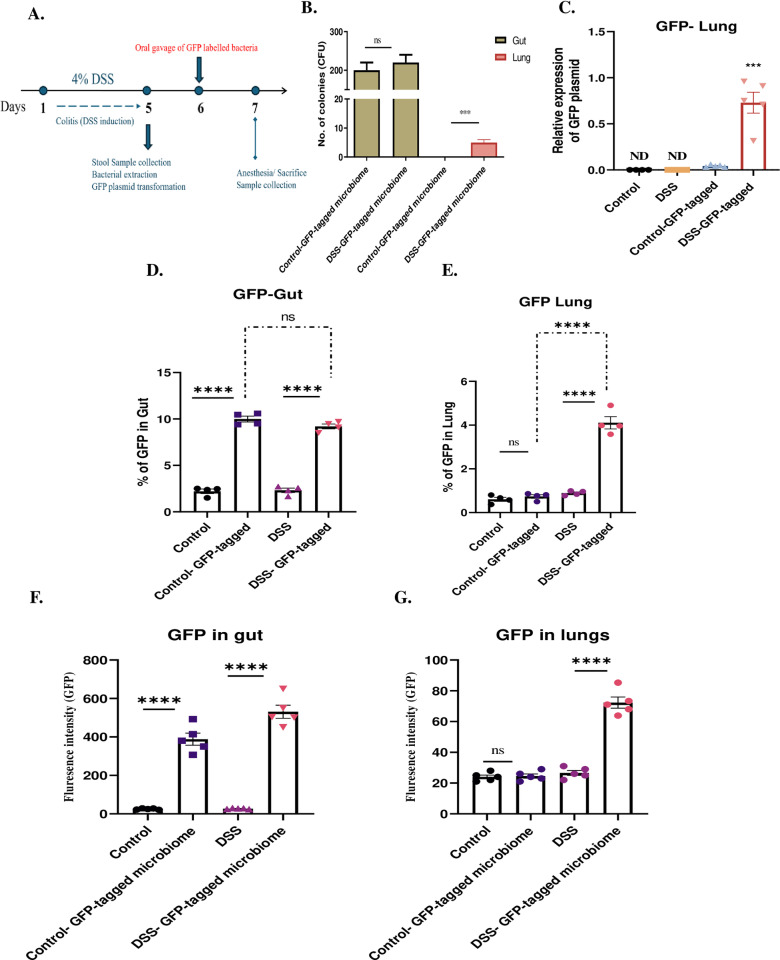
Presence of GFP plasmid in the lungs of DSS-induced colitis mice due to intestinal leakage. **(A)** Experimental design of the GFP-tagged fecal microbiome experiment, illustrating the timeline and procedure for administering GFP-tagged plasmid-bearing fecal microbiota to control and DSS-treated mice. **(B)** Colony formation on Ampicillin-containing agar plates from lung and gut samples of control and DSS-treated mice with GFP-tagged microbiome. **(C)** qPCR analysis of GFP plasmid DNA expression in lung tissues of control and DSS-treated mice with/ without GFP-tagged microbiome. **(D)** Flow cytometry analysis of GFP-positive cells in gut tissues of control and DSS-treated mice with/ without GFP-tagged microbiome. **(E)** Flow cytometry analysis of GFP-positive cells in lung tissues with/ without GFP-tagged microbiome. **(F)** Measurement of GFP levels in supernatants from gut and, **(G)** lung homogenates of DSS-treated mice with/ without GFP-tagged microbiome. Data representative from n = 5 mice in each group Results are presented as mean (± SEM) and were compared using an independent student’s t-test. *P < 0.05, **P < 0.01, ***P < 0.001, ****P < 0.0001 compared to control mice.

Another potential mechanism of gut-lung crosstalk is the translocation of bacteria from the gut to the lung. To investigate this, fecal microbiota was isolated from stool samples of control and DSS-treated mice. The isolated fecal bacteria were transformed with GFP tagged plasmids (pBTK503 and pBbB5a-GFP) and administered orally into both control and DSS-treated groups respectively on Day 6. After 24 hours, the mice were sacrificed as shown in (Fig 5A). From the lung and gut of the control and DSS mice, GFP tagged plasmid bearing fecal microbiota was isolated on Ampicillin containing agar plates and the colonies were counted and recorded. Colonies were observed on plates with lung and gut samples of DSS- GFP- tagged microbiome group, while colonies were observed only for the gut samples of control- GFP- tagged microbiome group (Fig 5B and S4A Fig), indicating bacterial translocation from gut to lung in the DSS- GFP- tagged microbiome group. To confirm this finding, qPCR was performed to measure the expression of GFP plasmid DNA in both control and DSS mice in presence or absence of GFP- tagged microbiome. Interestingly, a significant increase in GFP plasmid DNA was observed within the lungs of DSS-treated mice that received GFP- tagged microbiome (0.7 vs 0.04 log-fold; P** **< 0.001), compared to control mice group that received GFP- tagged microbiome (Fig 5C). GFP plasmid DNA was not detected in untreated control and DSS groups. This was confirmed on agarose gel as shown in (S4B Fig).

To further validate our findings, we used flow cytometry to detect GFP in the gut and lungs of both control and DSS-treated mice. In the gut (Fig 5D and S5A Fig), flow cytometry results showed a significant increase in GFP-positive cells in both control (10% vs 2.2%; P < 0.0001) and DSS-treated mice (9.2% vs 2.3%; P < 0.0001) that received the GFP- tagged microbiome compared to those that didn’t. However, in the lungs (Fig 5E and S5B Fig), the percentage of GFP-positive cells were significantly increased only in DSS-treated mice which received the GFP- tagged microbiome compared to those that didn’t receive (2.6% vs 0.8%; P** **< 0.0001) and to control mice that received the GFP- tagged microbiome (0.7%; P** **< 0.0001). These data support the idea that GFP- tagged microbiome translocate from the gut to the lungs in DSS-induced colitis.

Additionally, we detected a significant increase in GFP levels in the supernatants of both gut (Fig 5F) and lung (Fig 5G) homogenates from DSS-treated mice that received the GFP- tagged microbiome. These results together provide strong evidence for the translocation of bacteria from the gut to the lungs in a DSS-induced colitis model suggesting a significant role in the gut-lung inflammatory axis.

## 4. Discussion

**Fig 6:**
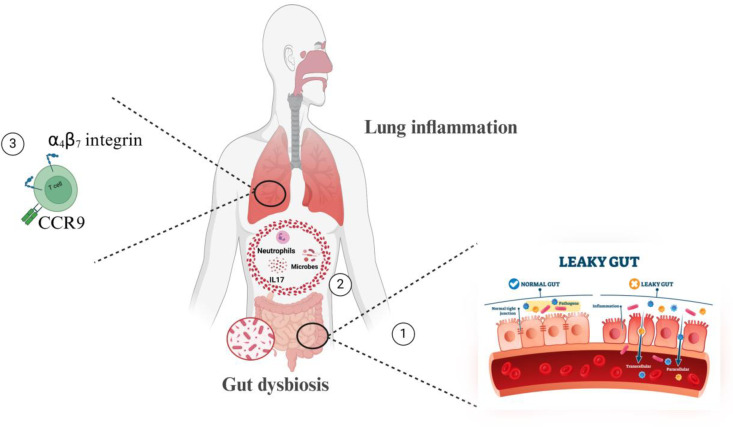
Mechanisms of colitis induced lung inflammation. Briefly, **(1)** gut dysbiosis, or an imbalance in the gut microbiota, can impact gut anatomy, physiology, and immune responses. Furthermore, intestinal inflammation can cause epithelial barrier disruption, leading to the **(2)** translocation of gut contents, including microbes, immune cells, and pro-inflammatory mediators, into the lungs, exacerbating inflammatory responses. Finally: immune cell mis-homing **(3)** occurs when immune cells expressing gut-homing receptors migrate from the gut to the lungs, contributing to inflammation and exacerbating conditions like asthma and COPD by disrupting the local immune environment in the lungs.

Recent studies have explored colitis-induced lung inflammation in DSS mice models, focusing on molecular pathways such as inflammasome activation and IL-6 signalling [22,36]. However, the mechanistic contributions from gut to the pulmonary inflammatory diseases are not well understood. Our research uniquely addresses this gap by elucidating the interplay between gut leakage, cytokine spillover (e.g., IL-17), microbial translocation, and immune cell trafficking (via α4β7 and CCR9) in driving pulmonary inflammation (Fig 6). This approach provides a comprehensive understanding of the gut-lung axis and highlights critical, underexplored pathways that could serve as therapeutic targets. By integrating molecular, immunological, and microbiological perspectives, our study offers a rational framework for addressing systemic inflammation in colitis, distinguishing it as a significant advancement in the field.

Gut leakage is characterized by increased intestinal permeability, followed by translocation of bacteria or bacterial products from the intestines to the mesenteric lymph nodes (MLN) and systemic organs, e.g., liver [37], spleen [38] and lungs [39,40]. Recent and previous studies have well-documented the disruption of tight junctions and increased gut permeability in DSS-induced colitis [41]. Our findings corroborate these observations, demonstrating a significant increase in orally administered FITC-dextran in serum and increased mRNA expression of gut leakage markers like Zonulin, LBP and IFABP in intestines of DSS-induced colitis mice due to highlighting the breaching of intestinal barrier integrity (Fig 1F–1G). In addition, we also observed an altered mRNA expression of tight junction proteins like ZO-1, Caludin-1 and Occludin-1 in the intestines of DSS-induced colitis mice (Fig 1I–1J) reflecting intestinal barrier dysfunction. This compromised gut integrity facilitates the translocation of microbial components, including LPS and peptidoglycans, into systemic circulation, [42]. Previous studies on DSS-induced colitis and its impact on distant organs such as the liver through gut barrier dysfunction demonstrated an elevated portal LPS levels [43]. This study supports the concept of systemic effects arising from gut inflammation, the “leaky gut” hypothesis. In consistent with the previous studies, our study demonstrates a significant lung inflammation, characterized by inflammatory cell infiltration, airway remodelling, and hyperresponsiveness associated with DSS-induced colitis. Moreover, elevated IL-17 levels in serum and BAL fluid implicate this cytokine as a central mediator of gut-lung crosstalk (Fig 2). IL-17 is known to promote neutrophil recruitment and exacerbate inflammatory responses [44], which was evident in the increased neutrophils observed in the lungs of DSS-treated mice (Fig 2E). Similar results were observed in DSS mice reported by Mateer et al, where they demonstrated that IL-6 plays a central role in neutrophil recruitment and pulmonary inflammation derived by bacteraemia [36]. Notably, while IL-4 and IL-13 levels were also mildly elevated, its role appears secondary to IL-17, suggesting a predominantly Th17-mediated mechanism in the GLA (Fig 3B and 3C). Additionally, Th1-related cytokines, particularly IFN-γ and TNF-α, also play a crucial role in the disease process, especially during the acute phase of inflammation [36,45]. These results highlight a potential interplay between Th17-driven neutrophilic inflammation and Th2-mediated eosinophilic activity, suggesting a complex immune dynamic in the lung inflammation associated with gut inflammation.

Growing studies observe a potential role of microbial translocation from gut to extra-intestinal tissues manifesting extra-intestinal diseases [15,16]. Therefore, we evaluated the bacterial translocation from the gut to the lungs using GFP-tagged microbiome in DSS induced colitis mice. Our data confirmed the presence of orally administered GFP-tagged microbiome in the lung tissues of DSS-treated mice (Fig 5), highlighting the critical role of gut leakage and its consequent microbial translocation in promoting lung inflammation. This novel finding provides direct evidence that microbial translocation is one of the pivotal drivers of the gut-lung inflammatory axis. Our results correlate well with previous studies that demonstrated bacteraemia in DSS-treated mice suggesting the potential role in manifesting extra-intestinal manifestations via bacterial translocation during colitis [46,47].

The existing literature indicates that gut-homing receptors play an essential role in maintaining intestinal homeostasis, as they ensure the precise recruitment of Th17 cells to the intestinal sites where they are most needed [48]. The expression of integrins like α4β7 and chemokine receptors such as CCR9 allows Th17 cells to migrate through the bloodstream and adhere to the vascular endothelium in the gut [49]. After reaching the lamina propria, these cells can exert their protective functions against pathogens and regulate the local immune responses to maintain homeostasis. This targeted migration is crucial because an overabundance of Th17 cells in the gut can lead to excessive inflammation and tissue damage, as seen in conditions like IBD [50]. In an EAE mice model, blocking the α4β7-integrin/MAdCAM-1 homing pathway not only limits intestinal Th17 cell infiltration but also significantly reduces EAE severity [51]. In another interesting study, the researchers highlighted the role of CCR9/ CCL25 signalling in allergic airway inflammation in an OVA mice model [52]. These findings underscore how disruption of normal gut-immune homeostasis can lead to inappropriate immune cell trafficking and subsequent development of extraintestinal diseases. Our study confirmed the upregulation of α4β7 and CCR9 (Fig 4) in DSS-treated mice lung tissues, suggesting that chronic intestinal inflammation alters immune cell trafficking, enabling gut-primed lymphocytes to migrate to the lungs. This mis-homing mechanism facilitates the outspread of immune responses from the gut to the lungs, amplifying local inflammation.

These findings have significant clinical implications for understanding and managing extraintestinal manifestations of IBD. Targeting the gut-lung axis through interventions that restore gut barrier integrity, modulate immune cell trafficking, and mitigate microbial translocation may provide novel therapeutic avenues for patients with IBD and its associated respiratory complications. For instance, therapies aimed at enhancing tight junction function or reducing gut dysbiosis may alleviate systemic inflammation and its downstream effects on the lungs. Furthermore, the identification of IL-17 as a key mediator in the GLA suggests that anti-IL-17 therapies, currently used in autoimmune diseases, may have the potential to address IBD-associated lung inflammation. The involvement of bacterial translocation highlights the need to explore probiotics or microbiota-targeted interventions to restore microbial homeostasis and mitigate intestinal barrier dysfunction to reduce systemic inflammatory responses. For example, probiotics like *Lactobacillus reuteri* has shown to induce expressions of intestinal tight junction proteins, reverse dysbiosis observed in IBD and modulate barrier function [53]. Despite our study re-establishes the gut-lung axis with robust evidence of bacterial translocation and mis-homing of immune cells from gut to lung in DSS-induced colitis, some limitations warrant consideration. The findings are derived from a preclinical mouse model, and their translation to human IBD patients requires further investigation. Additionally, while bacterial translocation was demonstrated, the specific bacterial strains and their role in pulmonary inflammation remain to be identified. Future studies should focus on characterizing the patrolling microbiota within gut-lung axis and exploring their potential as diagnostic biomarkers or therapeutic targets.

## Supporting information

S1 Fig Flow cytometry gating strategy. **(A)** Representative flow cytometry plots show the gating strategy for neutrophils and eosinophils, **(B)** Gut homing receptors, and **(C)** GFP-tagged fecal microbiome.
(DOCX)

S2 Fig Neutrophils and eosinophils in lungs of DSS mice. **(A)** Flow cytometry analysis of neutrophil and **(B)** eosinophil infiltration in lung tissues.
(DOCX)

S3 Fig Gut homing receptors in gut and lungs of DSS mice. **(A)** Flow cytometry analysis of gut homing receptors in gut and **(B)** lungs of control and DSS mice.
(DOCX)

S4 Fig GFP-tagged fecal microbiome experiment confirms microbial translocation to the lungs. **(A)** Colony formation on Ampicillin-containing agar plates from lung and gut samples of control and DSS-treated mice with/ without GFP-tagged microbiome; red arrows show bacterial colonies. **(B)** Agarose gel electrophoresis confirming the presence of GFP plasmid DNA in lung tissues of DSS-treated mice with/ without GFP-tagged microbiome.
(DOCX)

S5 Fig Flow cytometry analysis of GFP-tagged fecal microbiome in gut and lungs. **(A)** Representative flow cytometry plots show the difference between control and DSS group with/ without GFP-tagged- fecal microbiome treatment in gut, and **(B)** in lungs.
(DOCX)

S1 Raw images (PDF)
