# Supplementary figures and images for: The role of gut leakage and immune cell miss-homing on gut dysbiosis-induced lung inflammation in a DSS mice model
Source: PLoS One. 2025 May 28;20(5):e0324230. doi: 10.1371/journal.pone.0324230 (PMC12118880; doi:10.1371/journal.pone.0324230)

**Supplementary Fig. 1**

**A**

**
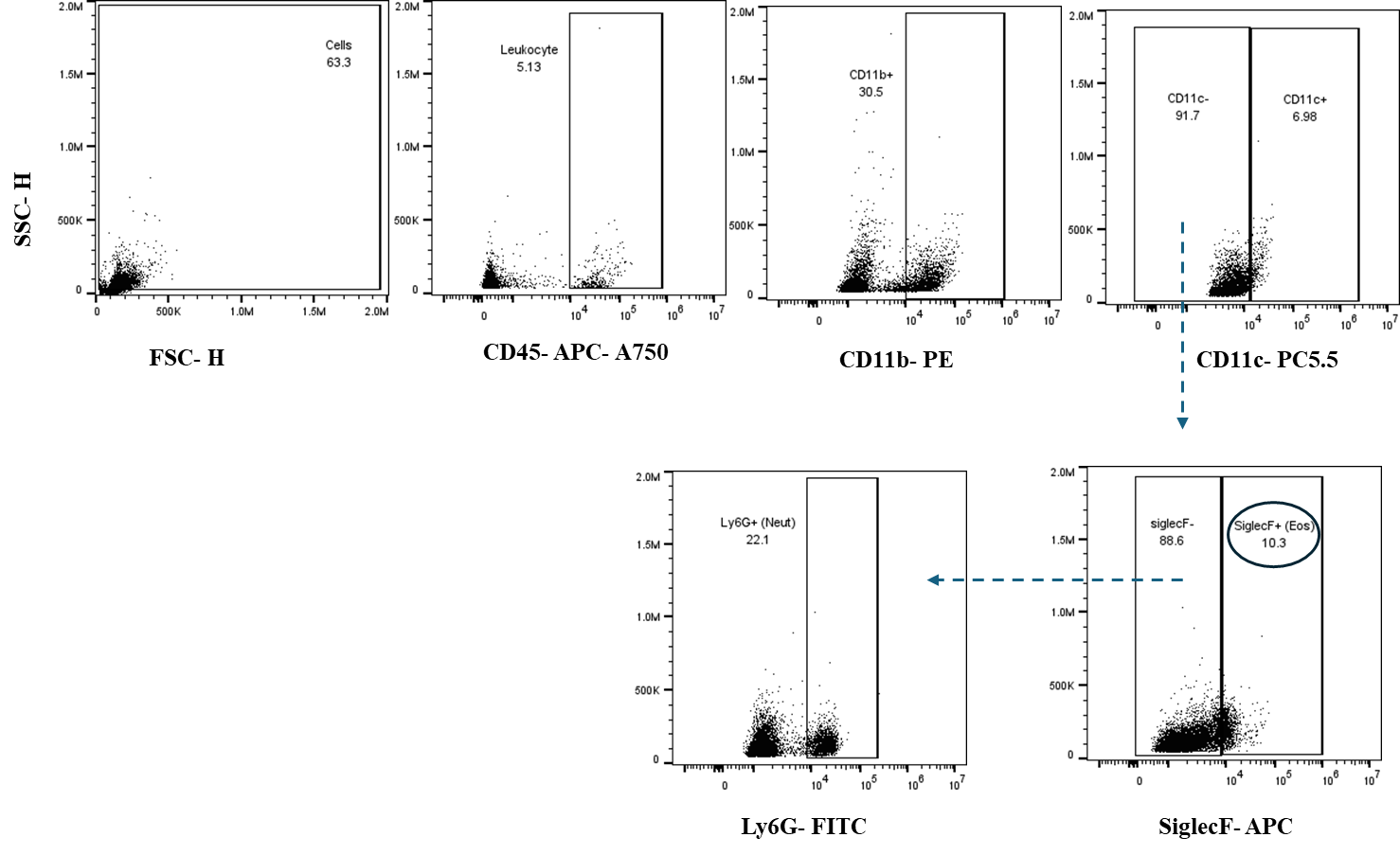
**


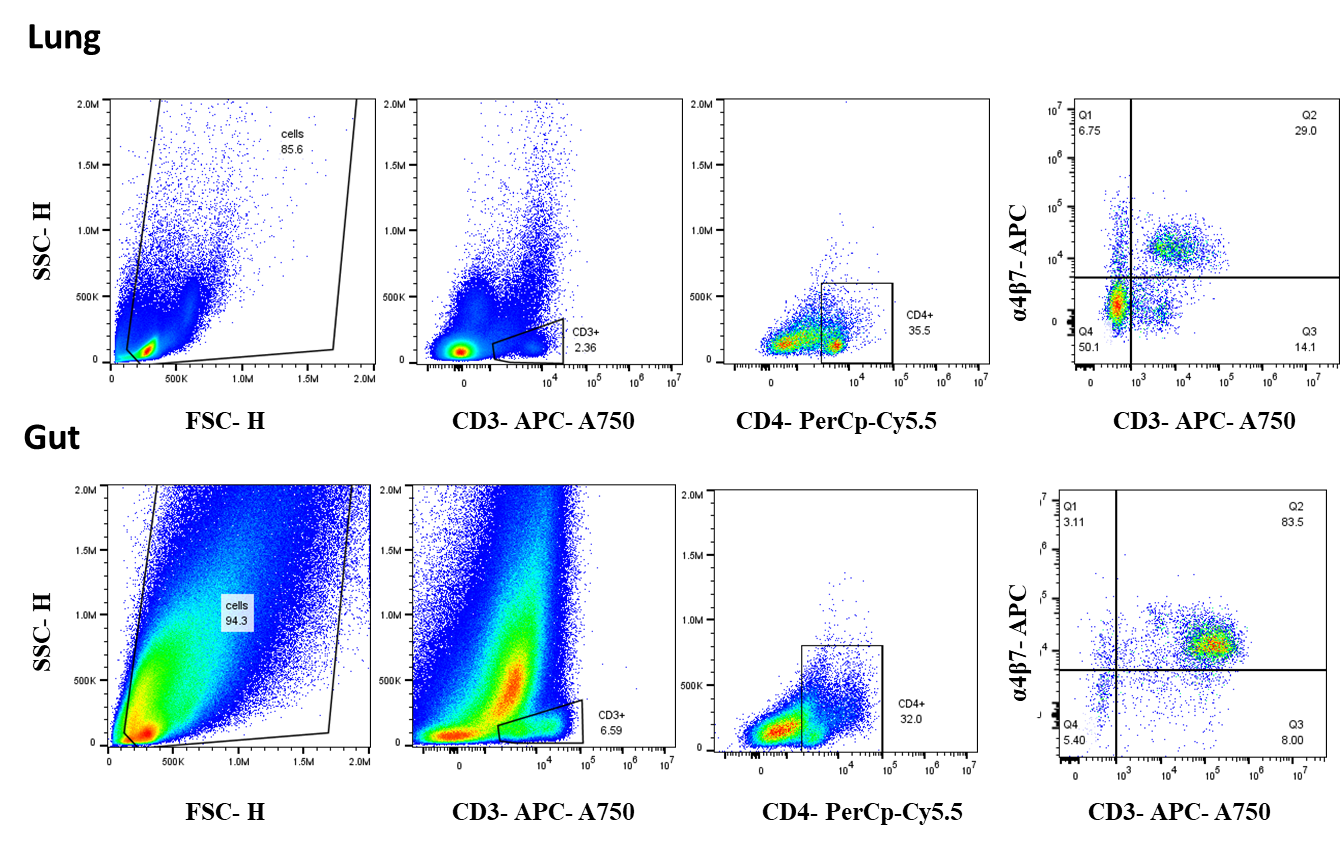
**B**

**C**


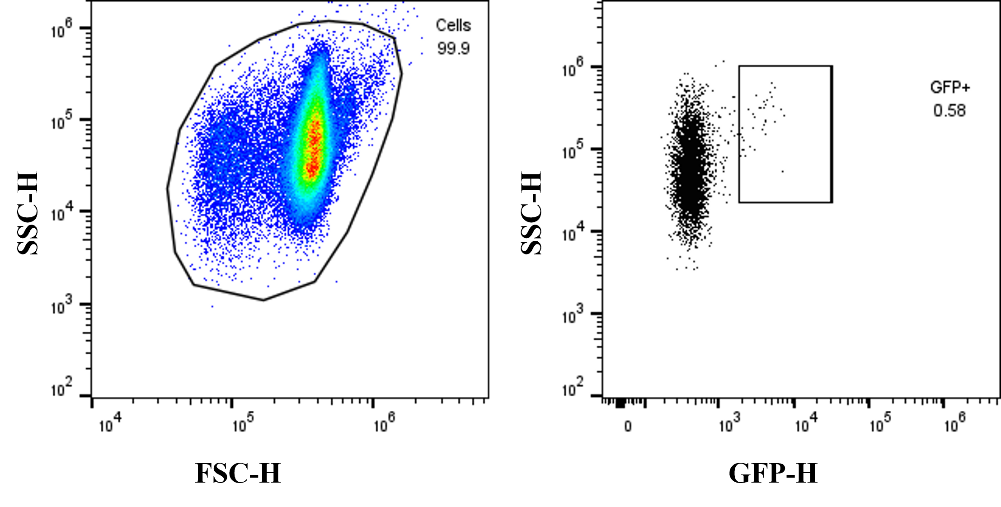

Supplement: S1 Fig — (A) Representative flow cytometry plots show the gating strategy for neutrophils and eosinophils, (B) Gut homing receptors, and (C) GFP-tagged fecal microbiome. (DOCX) [file pone.0324230.s001.docx]

**Supplementary Fig. 2**

**
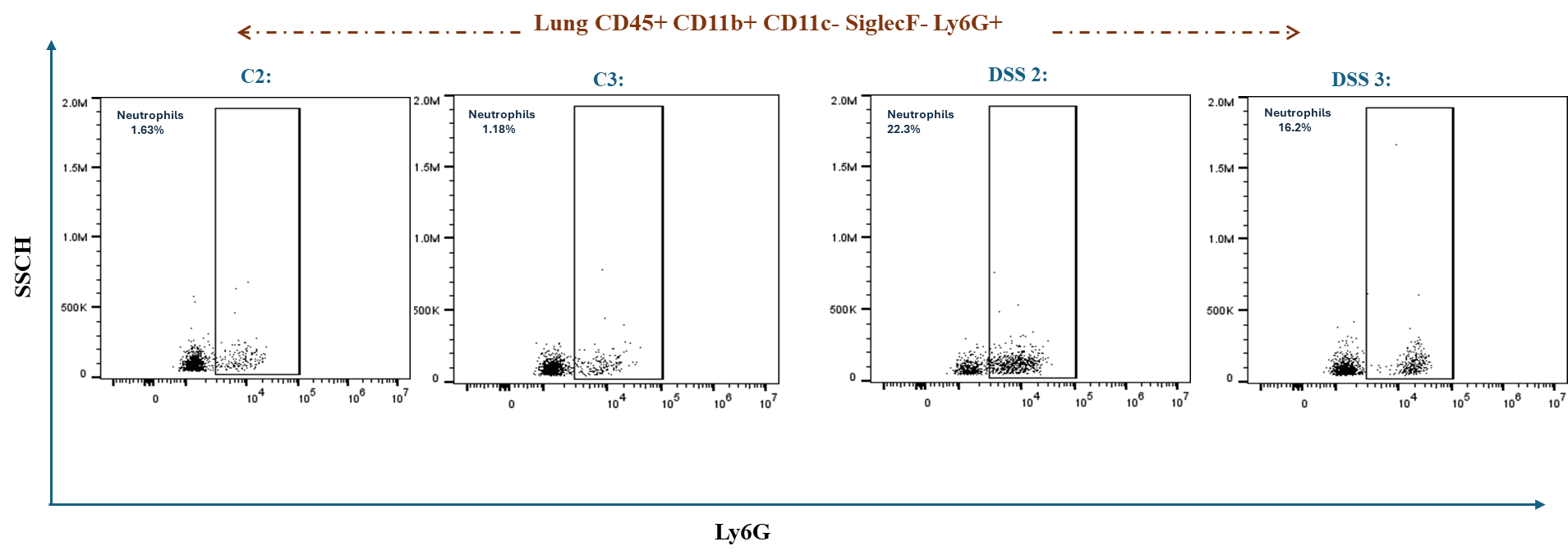
A**

**B**

**
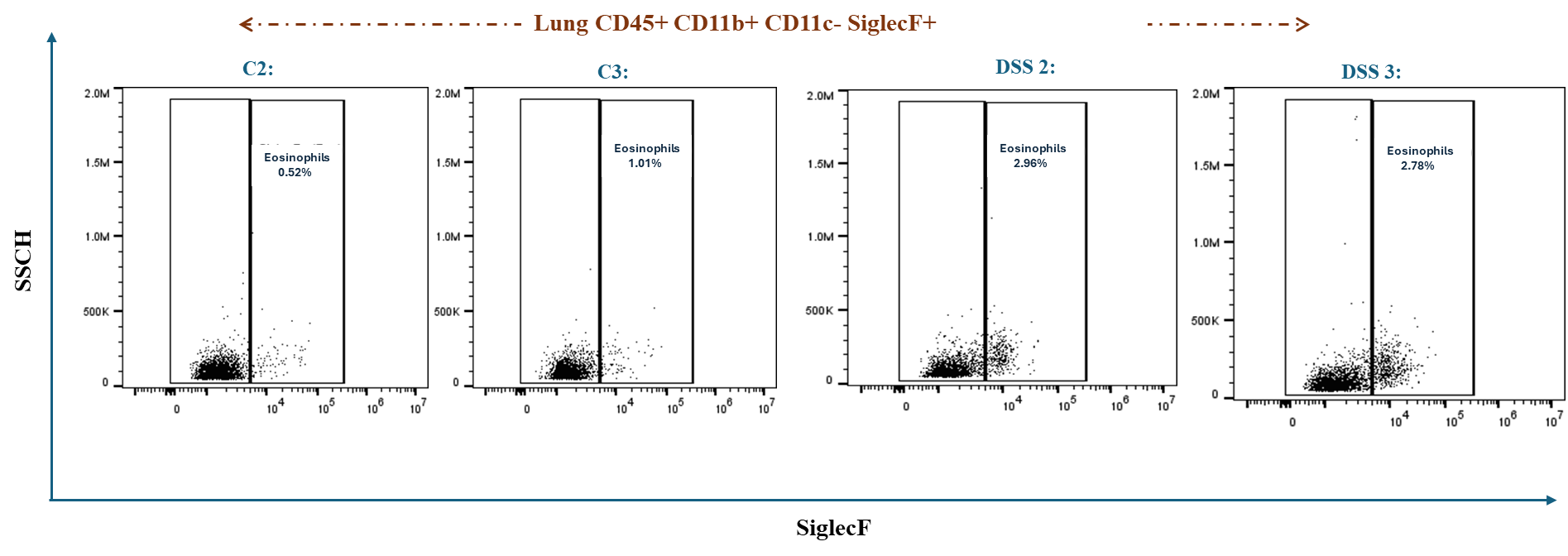
**

Supplement: S2 Fig — (A) Flow cytometry analysis of neutrophil and (B) eosinophil infiltration in lung tissues. (DOCX) [file pone.0324230.s002.docx]

**Supplementary Fig. 3**

**
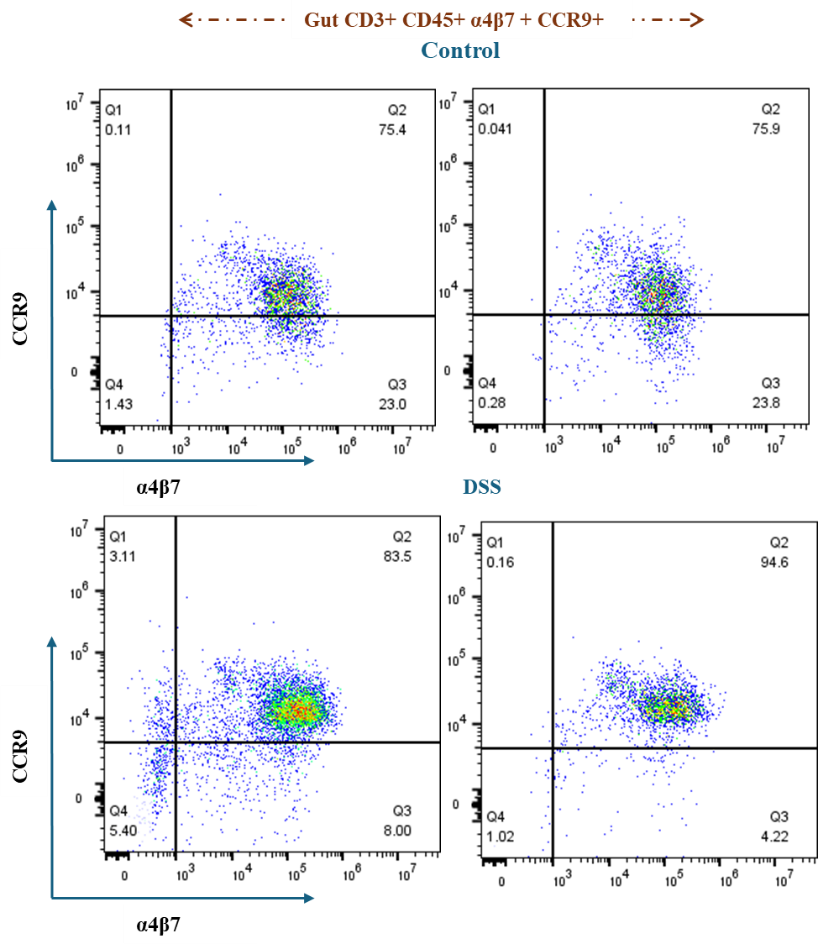
A**

**
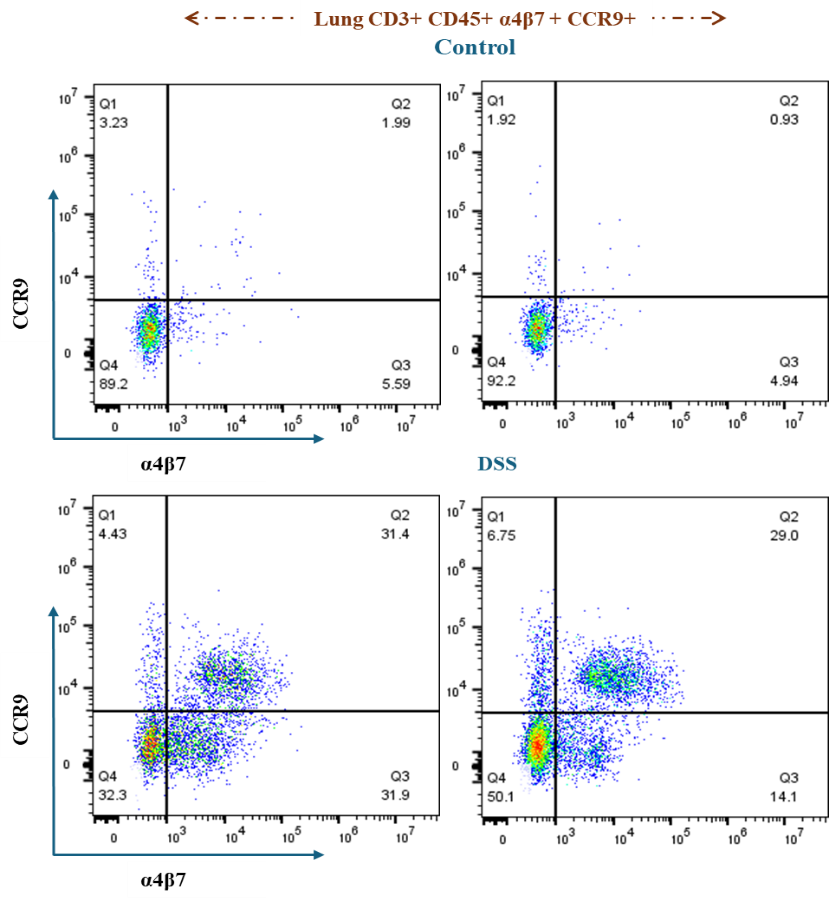
**

**B**

Supplement: S3 Fig — (A) Flow cytometry analysis of gut homing receptors in gut and (B) lungs of control and DSS mice. (DOCX) [file pone.0324230.s003.docx]

**Supplementary Fig. 4**

**A**


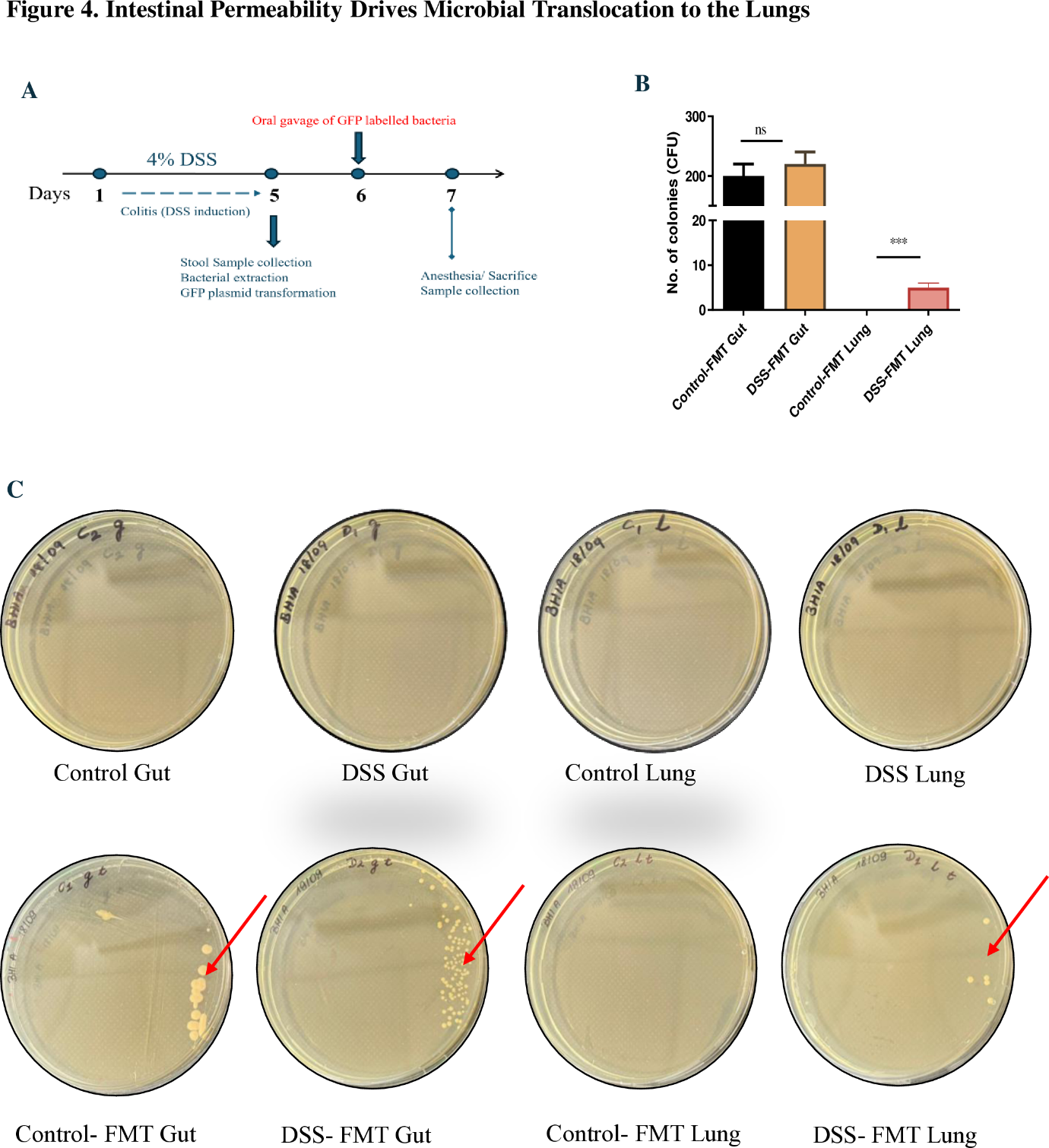


**B**

-ve Control

Control

DSS

Control- GFP-tagged

DSS-GFP-tagged


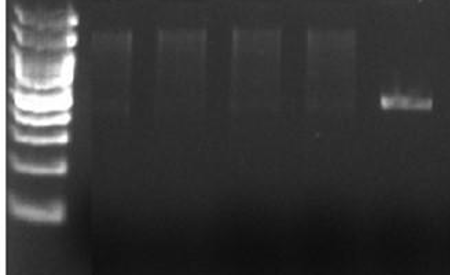


Lung

Supplement: S4 Fig — (A) Colony formation on Ampicillin-containing agar plates from lung and gut samples of control and DSS-treated mice with/ without GFP-tagged microbiome; red arrows show bacterial colonies. (B) Agarose gel electrophoresis confirming the presence of GFP plasmid DNA in lung tissues of DSS-treated mice with/ without GFP-tagged microbiome. (DOCX) [file pone.0324230.s004.docx]

### Figure A

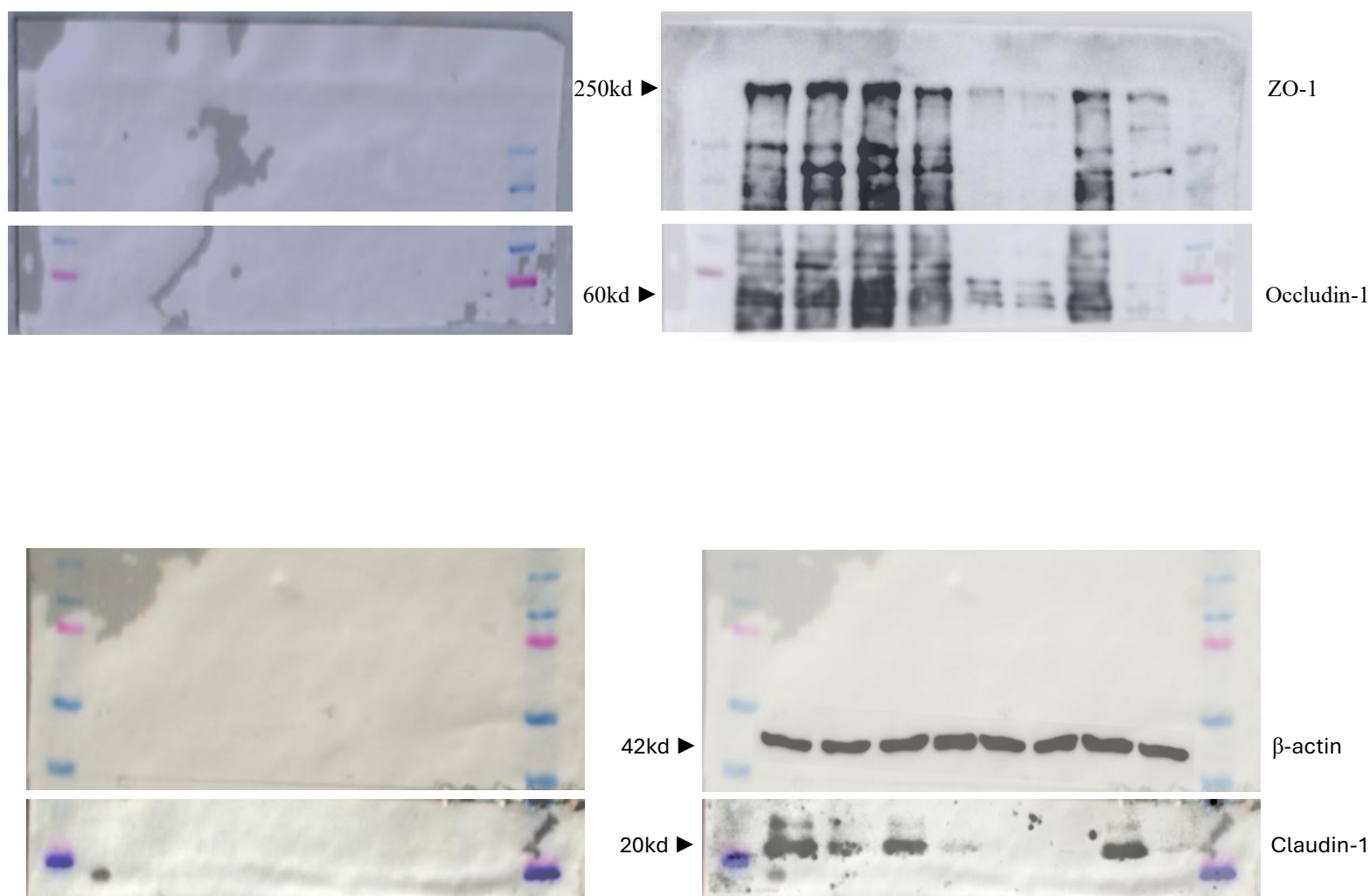

Supplement: S1 Raw images — (PDF) [file pone.0324230.s006.pdf]
