# Supplementary material for: The role of gut leakage and immune cell miss-homing on gut dysbiosis-induced lung inflammation in a DSS mice model
Source: PLoS One. 2025 May 28;20(5):e0324230. doi: 10.1371/journal.pone.0324230 (PMC12118880; doi:10.1371/journal.pone.0324230)
Supplement: S5 Fig — (A) Representative flow cytometry plots show the difference between control and DSS group with/ without GFP-tagged- fecal microbiome treatment in gut, and (B) in lungs. (DOCX) [file pone.0324230.s005.docx]

**Supplementary Fig. 5**

**A**

Gut:

**Control**

**Control-GFP-tagged microbiome**

**DSS**

**SSC-H**

**GFP-H**

**SSC-H**

**GFP-H**


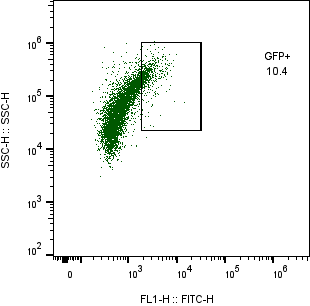


**GFP +ve**

**10.47**


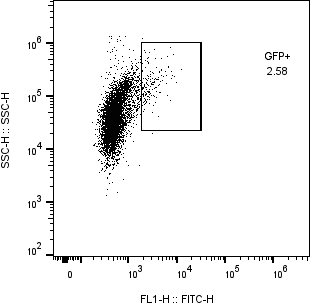


**GFP +ve**

**2.58**


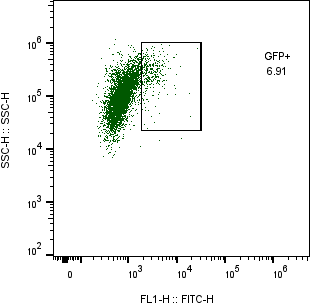


**GFP +ve**

**9.47**


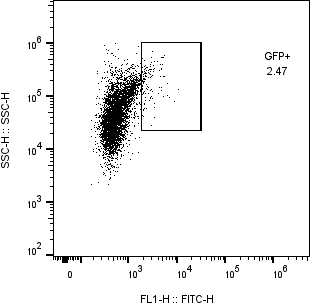


**GFP +ve**

**2.47**

**DSS-GFP-tagged microbiome**

**B**

Lung:


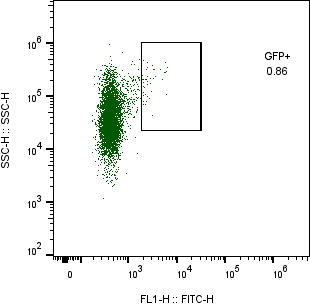


**GFP +ve**

**0.86**


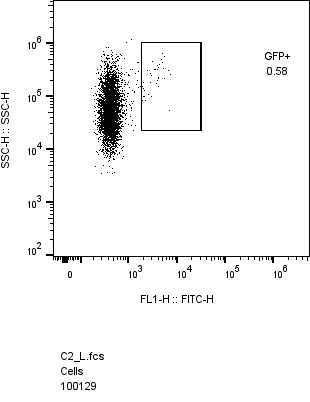


**GFP +ve**

**0.58**


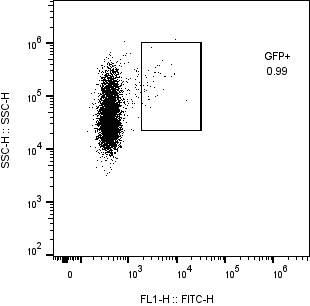


**GFP +ve**

**0.99**


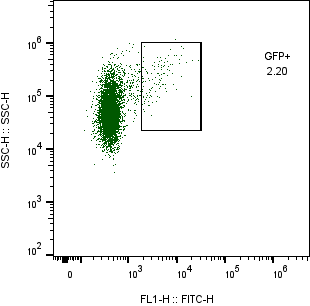


**GFP +ve**

**4.9**

**SSC-H**

**GFP-H**

**SSC-H**

**GFP-H**

**Control**

**Control-GFP-tagged microbiome**

**DSS**

**DSS-GFP-tagged microbiome**
